# Enhancing the Corrosion Resistance of Al–Cu–Li Alloys through Regulating Precipitation

**DOI:** 10.3390/ma13112628

**Published:** 2020-06-09

**Authors:** Jinjun Xu, Yunlai Deng, Jiqiang Chen

**Affiliations:** 1 Light Alloy Research Institute, Central South University, Changsha 410083, China; encourage@csu.edu.cn; 2 School of Materials Science and Engineering, Central South University, Changsha 410083, China; 3 State Key Laboratory of High Performance and Complex Manufacturing, Central South University, Changsha 410083, China; 4 School of Material Science and Engineering, Jiangxi University of Science and Technology, Ganzhou 341000, China; chenjiqiang@jxust.edu.cn; 5 Dongguan Eontec Co. Ltd., Dongguan 523662, China

**Keywords:** Al–Cu–Li, pre-strain, aging treatments, corrosion properties, microstructure

## Abstract

The influences of aging treatments on microstructures and the corrosion properties of an Al–Cu–Li alloy were investigated through an immersion test in intergranular corrosion (IGC) solutions, a potentiodynamic polarization test, and electrochemical impedance spectra (EIS), combined with scanning and transmission electron microscopy. The results demonstrated that the Al–Cu–Li alloy displayed outstanding comprehensive mechanical properties and IGC resistance after treating with pre-strain deformation and a double aging process (PDA). Both the PDA and pre-strain followed by creep aging (PCA) treatments significantly increased the number densities of T_1_ and θ’ precipitates in the grain interior. The increase in precipitates in the grain interior greatly reduced the Cu-rich precipitates on the grain boundaries and inhibited the formation of a precipitate-free zone (PFZ). The electrochemical characteristics of the Al–Cu–Li alloy were influenced by the precipitates in the grain interior and grain boundaries. The studied alloy gained high IGC resistance due to the refinement of its microstructure, and the main corrosion mode was intra-granular pitting corrosion; thus, the corrosion diffusion rate was slowed down.

## 1. Introduction

Lightness, long service life, and low environmental pollution are important topics in the aerospace and military industries [1]. In recent years, Al–Cu–Li alloys have attracted much attention in the aerospace industry owing to their display of outstanding properties, such as high specific strength, low density, and high damage tolerance performance [2]. Al–Cu–Li alloys usually maintain high corrosion resistance and outstanding mechanical properties simultaneously. Corrosion is considered to be a primary risk to the integrity of aviation equipment and results in great declines in service life. As far as aerospace applications are concerned, intergranular corrosion (IGC) shows a more detrimental corrosion morphology than intragranular corrosion in Al–Cu–Li alloys [3]. Research on improving IGC resistance is always an important topic since the occurrence of IGC limits the applications of engineering alloys.

The mechanical and corrosion properties of Al–Cu–Li alloys are related to intermetallic phases such as the T series (Al_2_CuLi, T_1_; Al_5_CuLi_3_, T_2_; Al_7_Cu_4_Li, TB), δ′ (Al_3_Li) and θ′ (Al_2_Cu) precipitates. The distribution and size of these precipitates are significantly influenced by thermo-mechanical processing [4,5]. The T_1_ phase is the primary strengthening precipitate in Al–Cu–Li alloys (such as the 2195, 2050 and 2090 alloys). This precipitate usually preferentially nucleates at dislocations, vacancies, and grain boundaries [6]. The number density of T_1_ phases is significantly increased by plastic deformation treatment before direct artificial aging. The high density of T_1_ precipitates has an effective pinning effect on dislocations, improving strength and toughness [7,8]. However, previous work [9,10] has indicated that the electrochemical potential of T_1_ precipitates differs from that of the matrix, leading to an increase in corrosion susceptibility for Al–Cu–Li alloys.

As indicated by Buchheit et al. [9], the preferential occurrence of intergranular or intragranular local corrosion depends on the position of the T_1_ precipitates. Grain boundaries with a high number density of T_1_ precipitates display high IGC susceptibility during the immersion of Al–Cu–Li alloys in NaCl solution. Ma et al. [10] indicated that the selective dissolution of T_1_ precipitates with high electrochemical activity leads to the development of local corrosion in the AA 2099-T83 alloy. In contrast, Yan [11] claimed that T_1_ precipitates nucleated and grew at the intragranular and intergranular regions in peak-aged AA2050 alloy. Such microstructure could balance the electrochemical behavior of the grain interior and grain boundaries, thus making the alloy immune to IGC. Li et al. [12] found that age-forming in the Al–3.7Cu–1.5Li–X alloy raised the number density of T_1_ precipitates and significantly diminished the size of these precipitates, thus improving the mechanical and corrosion properties of the alloy. 

To sum up, the existing literature shows that adjusting the aging process could improve the mechanical properties of Al–Cu–Li alloys through promoting T_1_ precipitation, but it would also cause more serious corrosion in the case of aging-treated alloys. However, it is still a challenge to balance mechanical properties and good corrosion resistance in Al–Cu–Li alloys. This work mainly centers upon the regulation and control of artificial aging technique processing on the microstructure and corrosion resistance of Al–Cu–Li alloys, aiming to establish a relationship between the evolution of microstructures and corrosion resistance, and then to optimize effective mechanical heat treatment processes in order to demonstrate the utilization potential of Al–Cu–Li alloys.

## 2. Experiments

### 2.1. Materials and Methods

The alloy ingots used in the experiment were prepared using a vacuum melting furnace. After homogenization, a sheet with 2 mm thickness was obtained by hot and cold rolling. Its nominal chemical composition is shown in Table 1. 

After solution heat treatment at 510 °C for 1 h followed by water quenching, all samples were separated into five groups. The different aging treatment procedures and the comprehensive mechanical properties of the studied alloy are given in our previous publications [8], and the relevant measurements are given in Table 2.

### 2.2. Corrosion Experiments

Corrosion tests were carried on differently aged samples. To reduce the influence of surface roughness on corrosion resistance, the corrosion samples were polished by 600, 2000 and 5000-grit sandpaper and then polished with 3 μm diamond paste. Next, the samples were put into 10% NaOH followed by 30% HNO_3_ solution for alkali and acid washing. Finally, the samples were cleaned with ethanol, washed with distilled water, and air-dried naturally. The other planes of the samples were sealed with paraffin. The corrosion tests were implemented by immersing the samples in a 57 g/L NaCl + 10 mL/L H_2_O_2_ solution (IGC solution) for 6 h, and the experimental temperature was kept at 35 (±2) °C by a thermostatic bath.

Following immersion testing, the corroded samples were thoroughly rinsed in deionized water, and then air-dried naturally. After carefully grinding with sandpaper and polishing with diamond paste, the cross-sections of the samples were inspected to determine their corrosion morphology and corrosion depth using an OLYMPUS GX71 metallographic microscope.

Electrochemical tests were performed by an electrochemical workstation (CHI660C, CHN) utilizing a three-electrode configuration cell. Two groups of parallel samples with surface area 1 cm^2^ were used for potentiodynamic polarization tests and electrochemical impedance spectra (EIS) measurements in 3.5 wt% NaCl solution at room temperature, respectively. The potentiodynamic scanning curves were tested, and the potential scanning speed was 1 mV/s. The EIS measurements were carried out with a sinusoidal potential of amplitude which was limited to 10 mV, and the frequency ranged from 10^–2^ to 10^5^ Hz.

### 2.3. Microstructure Characterization

After the grinding and polishing procedures, the distribution of intermetallic particles (IMPs) on the sample surface was characterized by a ZEISS EVO M10 scanning electron microscope (SEM). The intermetallic particles were measured using an energy dispersive spectrometer (EDS) attached to the SEM. Electron back-scattered diffraction (EBSD) measurements were conducted to characterize the grain structure of the alloy. The specimen was polished with 10% HClO_4_ and 90% C_2_H_5_OH solution at 20 V, and then the EBSD photographs were obtained by the same scanning electron microscope equipped with an OXFORD EBSD detector. For the preparation procedures for the transmission electron microscopy (TEM) samples and the method of observing transmission in microstructure, refer to previous publications [8].

## 3. Results

### 3.1. Corrosion Tests

**Figure 1:**
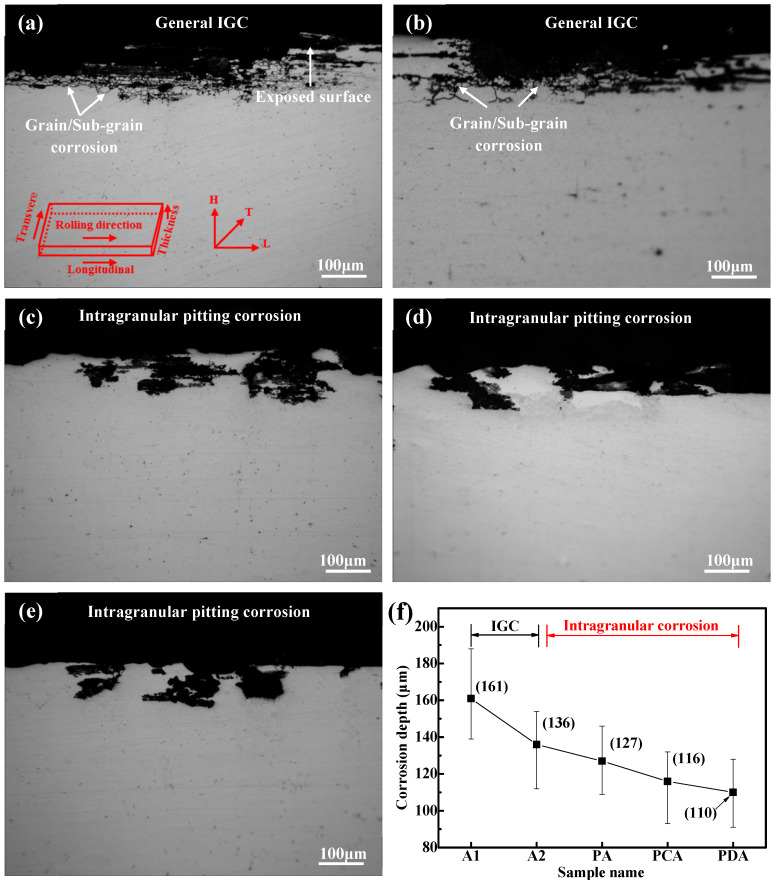
Representative cross-sectional corrosion morphologies of the five aged samples immersed for 6 h in IGC solution, representing: (**a**) A1, (**b**) A2, (**c**) PA, (**d**) PCA, (**e**) PDA; (**f**) Corrosion depth. Abbreviations: IGC, intergranular corrosion.

The samples treated with different aging treatments showed different corrosion morphologies after immersion in the IGC solution for 6 h, as shown in Figure 1. The samples treated by direct artificial aging (A1 and A2) exhibited high IGC, and propagated along the grain boundary, as shown in Figure 1a,b. Interestingly, almost no IGC was observed in the pre-strained samples (PA, PCA, PDA), and the corrosion morphologies took the form of intragranular pitting corrosion, as shown in Figure 1c–e. It is worth noting that intragranular corrosion is where subsurface pits occur. The propagation rate of the corrosion damage was determined by the corrosion depth of the cross-section of the corrosive sample. The measurement of mean and maximum corrosion depth is an important method for evaluating the corrosion defects of Al–Cu–Li alloys. The corrosion depth results for the alloy are shown in Figure 1f. It is obvious that the mean and maximum corrosion depths of the pre-strained samples were significantly lower than those of the direct artificially aged samples. In particular, the PDA (110 μm) and PCA (116 μm) samples reached lower corrosion depths compared to the other samples. Therefore, Al–Cu–Li alloys treated via different aging processes displayed various corrosion depths and morphology. 

### 3.2. Potentiodynamic Polarization Tests

**Figure 2:**
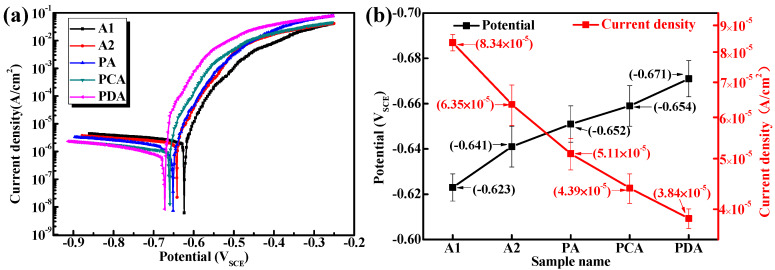
(**a**) Potentiodynamic polarization curves of the five aged samples during immersion in 3.5 wt% NaCl solution; (**b**) The values of corrosion potential (E_corr_) and corrosion current density (I_corr_).

Figure 2 shows the potentiodynamic polarization curve of the five aged samples after soaking in borate buffer 3.5 wt% NaCl solution for 600 s. As shown in Figure 2a, all the polarization curves exhibit a relatively smooth current platform in the cathodic region, which indicates the reaction of oxygen reduction. When the potential increases to the corrosion potential, the current density increases noticeably along with the increase in potential. It should be noted that current density increases to a certain extent, although while the potential increases continuously, the current density changes less, illustrating the existence of an oxide film. The corrosion potential (E_corr_) and corrosion current density (I_corr_) can be obtained by extrapolating the linear Tafel segments of the polarization curves in Figure 2a, and the results are shown in Figure 2b. On the basis of the results shown in Figure 2a, the values of E_corr_ for the A1 and A2 samples are significantly more positive than those of the pre-strained samples (PA, PCA, PDA). The E_corr_ of the A1 sample is the most positive with −0.623V_SCE_, with −0.641V_SCE_ for the A2 sample and −0.652V_SCE_ for PA, compared with the more negative results of PCA with −0.654V_SCE_ and PDA with −0.671V_SCE_. According to Faraday’s law, I_corr_ can be used as a dynamic factor to characterize the electrochemical corrosion rate of samples. PDA has the lowest I_corr_ compared to the other four samples, illustrating that the PDA sample had the slowest corrosion propagation rate, and this result agrees well with the result of the corrosion tests.

### 3.3. EIS Measurements

**Figure 3:**
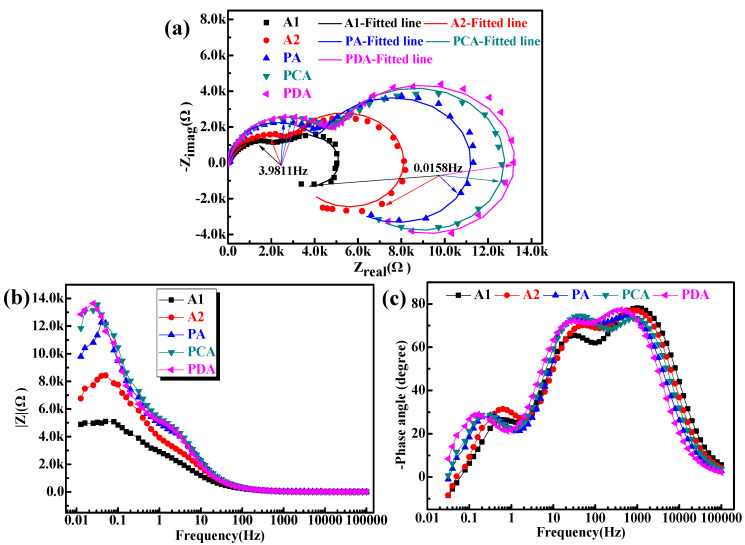
Nyquist plots (**a**) and corresponding Bode plots (**b**,**c**) of the five aged samples during immersion in 3.5 wt% NaCl solution.

Figure 3 shows the EIS of the five aged samples. The Nyquist plots of the alloy consist of two capacitive reactance arcs and an inductive arc, as shown in Figure 3a. 

In the high-frequency range, all EIS spectra displayed a capacitive arc illustrating the existence of an oxide film. The capacitive arcs at intermediate frequencies related to pitting corrosion. Moreover, the existence of low-frequency inductive arcs was related to the destruction of the oxide film and corrosion. Generally, the diameter of the Nyquist loop is a directive on the corrosion process for a sample surface under activation control. The increased diameter indicates that the alloy has good corrosion resistance. Furthermore, the magnitude of the capacitive arc mode is positively correlated with the corrosion resistance, while the larger the mode value (as shown in Figure 3b), the greater the reaction resistance and the slower the dissolution rate of the anode. According to the results of the EIS measurement, the PDA sample had the best corrosion resistance, which is in line with the IGC results.

**Figure 4:**
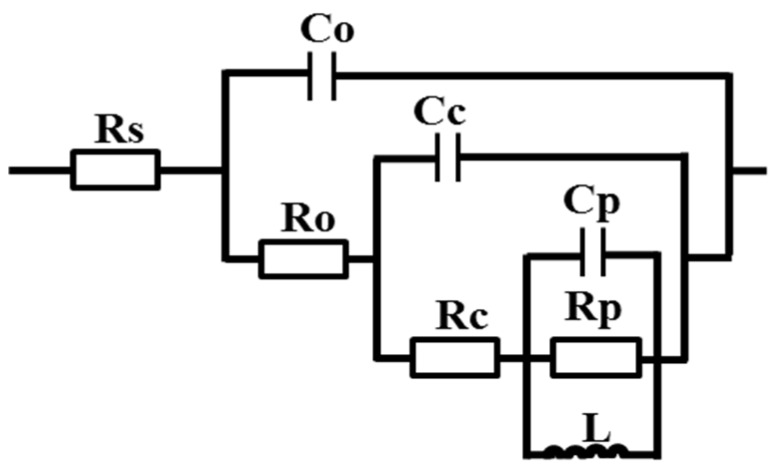
The equivalent electrical circuit (EEC) used to fit the electrochemical impedance spectra (EIS) test data.

To quantitatively analyze the experimental data and better explore the corrosion sensitivity of the Al–Cu–Li alloy, the EIS spectra were fitted to the equivalent electrical circuit (EEC) using the ZSimpwin software, as shown in Figure 4. The fitting curves are shown in Figure 3a, and the fitting results agree well with the experiment. The physical meaning of the EEC elements can be expressed as follows: R_s_, R_o_, and R_c_ represent electrolyte solution resistance, the resistance of the oxide film and corrosion products, and charge transfer resistance at the active corroded surface, respectively. C_o_ and C_c_ represent the capacitance of the oxide film with the origin surface and the capacitance of the new interface originating from pitting, respectively. R_p_, C_P_, and L represent the pseudo-resistance, capacitance, and inductance of the breakdown of the oxide film, respectively. The fitted values of the parameters of the EIS spectra are shown in Table 3. The results of the fitted values in Table 3 show that the value of R_c_ gradually increases in the order of A1, A2, PA, PCA, and PDA. The increase in R_c_ indicates a high resistance to active dissolution in the PCA and PDA samples.

### 3.4. The Second Phase and Grain Structure

**Figure 5:**
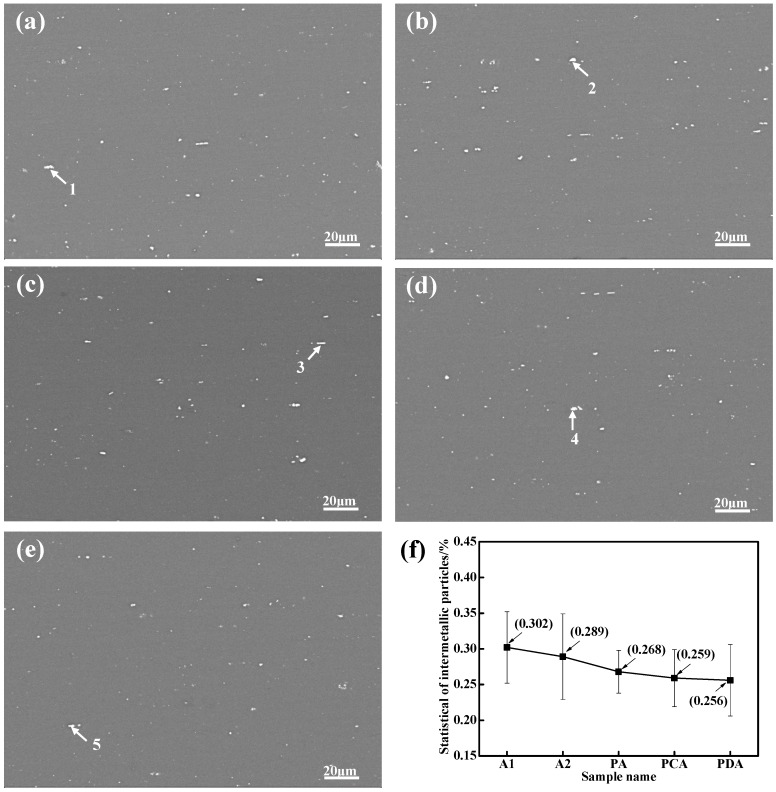
The scanning electron microscopy (SEM) micrographs of intermetallic particles in the five aged samples: (**a**) A1, (**b**) A2 (**c**) PA, (**d**) PCA, (**e**) PDA; (**f**) Area fraction of intermetallic particles (IMPs).

Figure 5a–e shows the SEM micrographs of the five aged samples, revealing that residual IMPs were indeed present in the samples. The area fraction of the IMPs for these five aged samples is shown in Figure 5f. According to the statistical results, the IMPs of the five samples showed little variation. The chemical compositions of the typical intermetallic particles in the five groups of aged samples are listed in Table 4. As Table 4 shows, these particles were all Al–Cu–Fe intermetallic. 

**Figure 6:**
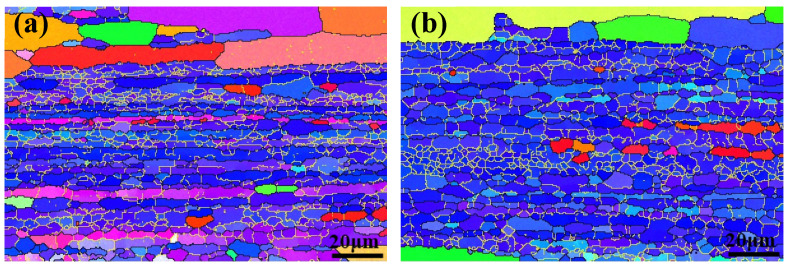
Electron back-scattered diffraction (EBSD) images of the specimens treated by the different aging treatments: (**a**) A1; (**b**) PCA.

Figure 6 shows the crystallographic orientation distribution of the examined region in Euler’s colors, with black lines and yellow lines indicating high-angle grain boundaries (HAGBs, θ ≥ 10°) and low-angle grain boundaries (LAGBs, 2° < θ < 10°), respectively. It is worth noting that EBSD tests were carried out on these five groups’ specimens, but because the EBSD structure of all samples is similar, only the EBSD pictures of A1 (Figure 6a) and PCA (Figure 6b) are listed here as representative. According to the above EBSD test, a large number of subgrains were retained in the five specimens. The T_1_ phase was easily enriched and precipitated on LAGBs. According to previous research [8], neither number density nor the average diameter of the T_1_ precipitate on the LAGB of pre–strained samples (PA, PCA, PDA) was less than those of direct artificially aged samples (A1, A2). In addition, the number density of T_1_ precipitates in the grain interior increased in the order of A1, A2, PA, PCA, and PDA, while the average diameter decreased gradually.

**Figure 7:**
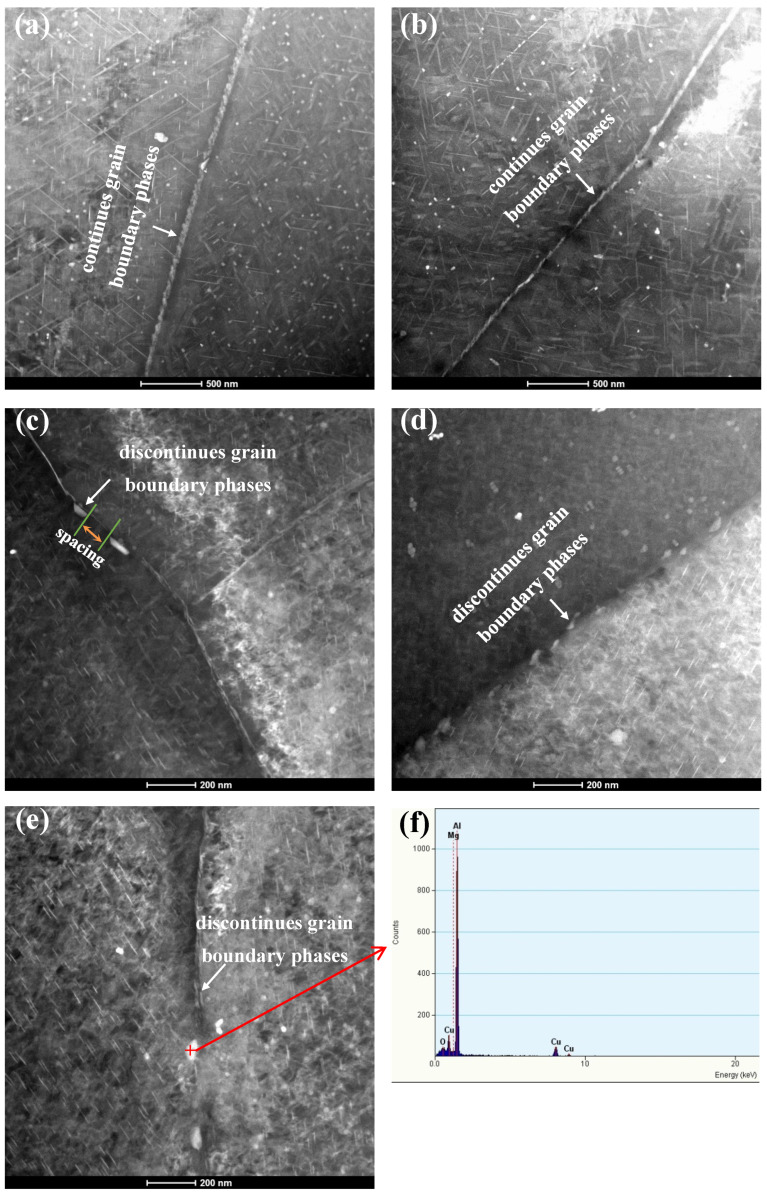
TEM micrographs of the five aged samples: (**a**) A1, (**b**) A2, (**c**) PA, (**d**) PCA, (**e**) PDA; (**f**) Energy dispersive X-ray (EDX) elemental maps of the framed area in (**e**).

Figure 7a–e shows the TEM morphology of the HAGBs under different aging conditions. In direct artificially aged samples (A1, A2), a large number of coarse phases were continuously distributed on the HAGB and the existence of a precipitate-free zone (PFZ) was indicated, as shown in Figure 7a,b. For the pre-strained samples (PA, PCA, PDA), Figure 7c–e shows that the number density of precipitates on the HAGB was obviously reduced and presented intermittent distribution, and that there were not obvious PFZs appearing near the HAGB. The chemical compositions of the typical coarse precipitates in the five aged samples were evaluated, and the results show that these precipitates were rich in Cu, as shown in Figure 7f. 

## 4. Discussion

According to the above experimental results, the aging treatment did not change the intermetallic particles and grain structure of the Al–Cu–Li alloy but significantly varied its precipitation behavior. 

During direct artificial aging treatment, solute atoms prefer to cluster on grain boundaries to decrease strain energy [13]. Therefore, in the A1 and A2 samples, a large number of coarse Cu-rich phases were continuously precipitated on HAGBs. Compared with HAGBs, LAGBs were generally composed of a series of dislocations, which are typically more likely to form T_1_ precipitates in Al–Cu–Li alloys [8,13]. The rapid growth of precipitates on the grain boundaries consumes many nearby atoms, resulting in a PFZ of a certain width. There were few nucleation points in the matrix, and the number of precipitates (T_1_ and θ′) in the grain interior was lower. For an analysis of the finding that PA, PCA and PDA treatments promote the precipitation of a larger number of T_1_ phases in the grain interior, refer to our previous publications [8]. A large number of intragranular precipitates were precipitated in the pre-strained samples, inevitably leading to the depletion of atoms from the matrix. In addition, the dislocations in the matrix introduced by the pre-strain hindered the diffusion of atoms to the grain boundaries. Therefore, the precipitates at the grain boundaries (including LAGBs and HAGBs) in the pre-strained samples were greatly reduced, and the formation of a PFZ was inhibited [14,15]. According to our previous publications [8], this excellent structure can make the alloy acquire outstanding mechanical properties. 

**Figure 8:**
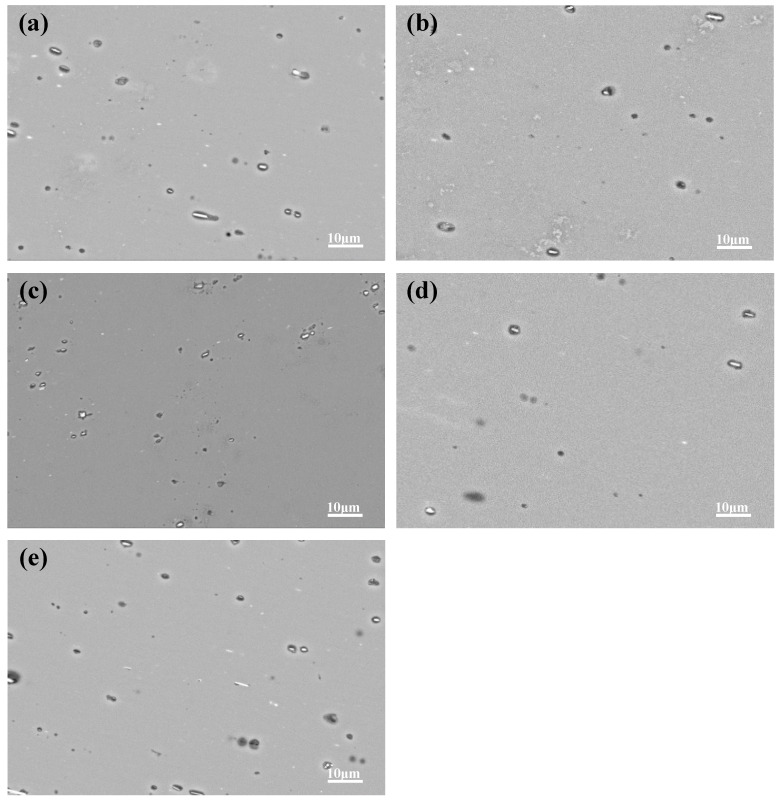
Representative corrosion surface morphologies of the five aged samples immersed for 1 min in IGC solution representing: (**a**) A1, (**b**) A2, (**c**) PA, (**d**) PCA, (**e**) PDA.

Since IMPs are usually inherent in the production of Al–Cu–Li alloys, the solution and aging treatment failed to dissolve them. The corrosion behavior of Al–Cu–Li alloys is closely related to the electrochemical inhomogeneity caused by IMPs [16,17,18,19]. The results of previous studies [18,19] noted that anodized IMPs introduce defects in the anodic film, leading to the preferential initiation and diffusion of corrosion. Figure 8 shows representative SEM micrographs of the corrosion surface of our five aged samples after immersion in corrosion solution for 1 min. The cavities and trenches observed at the peripheries of the IMPs were caused by the preferential dissolution of the surrounding anodic matrix [16,17,18,19]. With the prolongation of corrosion time, the priority corrosion area exposes the aluminum matrix to the fresh surface continuously, leading to the gradual deepening of the corrosion degree. However, the corrosion propagation paths of different aging systems are different. 

**Figure 9:**
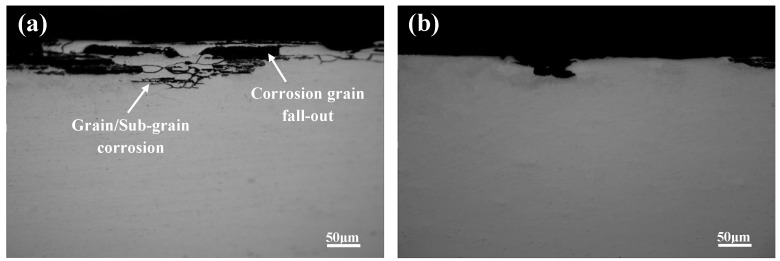
Representative cross-sectional corrosion morphologies of the five aged samples immersed for 1 h in IGC solution representing: (**a**) A1, (**b**) PDA.

Figure 9 shows the SEM morphology of the corrosion surface of the alloy after immersion in corrosion solution for 1 h. Obvious IGC occurred in the direct artificially aged samples (as shown in Figure 9a), while intragranular pitting corrosion occurred in pre-deformed samples (as shown in Figure 9b). In the direct artificially aged samples, the grain boundaries were ornamented with continuous Cu-rich precipitates, the corrosion potential of which was positive compared with the surrounding aluminum matrix; these acted as a sacrificial anode during the corrosion process. The continuous intergranular phases and the existence of a PFZ provided a convenient channel for electron transfer, which could expedite corrosion speed. This is consistent with the electrochemical test results: the A1 and A2 samples had a lower R_C_ and higher I_corr_ compared with the pre-strained samples. The above factors led to a great susceptibility of the A1 and A2 samples to IGC, and the corrosion depth of samples was greater in the same corrosion time [18]. 

The change of the corrosion morphology of the pre-deformed samples was related to the high density of T_1_ precipitates in the matrix. The corrosion potential of the T_1_ precipitates was more negative than that of the Al matrix. In an immersion test, Li in T_1_ precipitates is selectively dissolved and the noble element Cu becomes enriched; then, the Cu-rich residue turns into a cathode, resulting in the anodic dissolution of the nearby matrix [6,15,20,21]. This is similar to the effect of the θ′ precipitates promoting the anodic dissolution of the matrix, which usually causes homogeneous corrosion in the grain interior [12,19].

Moreover, the high number density of T_1_ precipitates in the grain interior combined with the depletion of matrix Cu resulted in the corrosion potential of the matrix becoming more negative. This is consistent with the trend in the value of E_corr_ in Figure 2a. The value of E_corr_ is only a qualitative parameter and cannot accurately indicate information about the number and size of pits, let alone the degree of damage caused by pitting. In addition, the number density of intergranular precipitates decreased, causing the corrosion potential of the grain boundary to become more negative [22]. Therefore, the difference in the electrochemical behavior of the grain boundaries and grain interior reduced to a degree at which IGC hardly ever occurs, thereby resulting in a significant elevation in IGC resistance [13,23,24,25,26]. Because of the negative corrosion potential of the intragranular precipitates, the matrix became more susceptible to corrosion, which is in line with the intragranular pitting corrosion of the pre-strained samples [3,13]. The distance between the precipitates provided a good condition for corrosion diffusion. In the pre-deformed samples, the high number density of the precipitates narrowed the effective corrosion area in the matrix, which effectively slowed down the corrosion diffusion rate. In fact, the value of R_o_ was confirmed by the resistance to the corrosion pit. The rising value of R_o_ indicated that the corroded area on the preformed samples was smaller than on the direct artificially aged samples [18].

**Figure 10:**
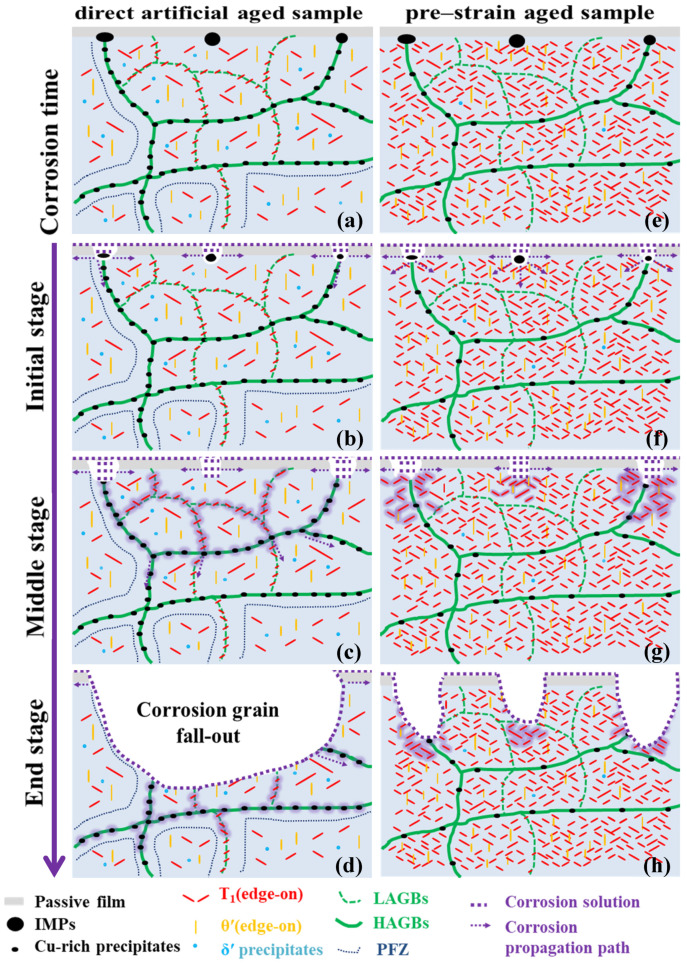
Schematic diagrams of corrosion process in Al–Cu–Li alloy: (**a**–**d**) direct artificially aged sample; (**e**–**h**) pre-strain-aged sample; (**a**,**e**) typical microstructure of samples; (**b**,**f**) initial stage of corrosion; (**c**,**g**) middle stage of corrosion; (**d**,**g**) end stage of corrosion.

Figure 10 schematically illustrates the corrosion process of the samples under different aging treatments immersed in the corrosive solution. Figure 10a,e show the typical microstructure images of the direct artificially aged and pre-strained samples, respectively. IMPs rich in Cu and Fe wreck the integrity of the oxide film and promote the preferential dissolution of the surrounding matrix, which leads to local corrosion and the formation of cavities. The corrosion solution contacts the alloy interface through the cavity, which leads to corrosion and then propagates along the bottom of the cavity (as shown in Figure 10b,f). The characteristics of grain boundary precipitation in the direct artificially aged samples mean that the corrosion can easily spread along the grain boundary and form a network structure at the bottom of the cavity, a phenomenon typical of IGC (as shown in Figure 10c). PDA treatment can increase the density of precipitates in the interior of the grain and reduce precipitates at the grain boundary, which makes the alloy exhibit intragranular pitting corrosion (as shown in Figure 10g). With the extension of corrosion time, a large number of corrosion products were produced at the grain boundary of the direct artificially aged samples, which forced the corrosion area to separate from the matrix and form a large-area cavity. This provided a good condition for the next IGC (as shown in Figure 10d). In the pre-strained sample, the precipitates in the interior grains were uniform and dense, which promoted the uniform corrosion of the alloy and effectively slowed down the corrosion rate; the decrease in precipitation spacing was not conducive to corrosion diffusion, so the corrosion spread slowly around the cavity (as shown in Figure 10h).

## 5. Conclusions

In summary, the microstructure evolution and corrosion properties of Al–Cu–Li alloys after treatment with different aging processes were investigated. On the basis of this study, these main conclusions were drawn:

(1) Compared with direct artificially aged samples, the pre-strain-aged sample (PA) significantly increased the number density of T_1_ and θ′ precipitates in the grain interior and inhibited the formation of a PFZ.
(2) PDA and PCA can further enhance the number density of intragranular precipitates and significantly decrease Cu-rich precipitates on grain boundaries (including LAGBs and HAGBs).
(3) Microstructure can make the alloy acquire outstanding comprehensive mechanical properties and IGC resistance; the main corrosion mode is transferred to intragranular pitting corrosion, which reduces corrosion depth. The high number density of precipitates in the grain interior narrows the effective corrosion area in the matrix, which effectively slows down the corrosion diffusion rate.

## Figures and Tables

**Table 1:** Nominal composition (wt%) of the Al–Cu–Li alloy.

Cu	Li	Mg	Ag	Zr	Si	Fe	Al
4.01	1.13	0.37	0.32	0.12	0.04	0.05	Bal

**Table 2:** Aging treatment procedures and mechanical properties of the Al–Cu–Li alloy.

Heat Treatment	Pre–Strain	Aging Treatment	Treatment Code	YS/MPa	TS/MPa	Elongation
510 °C/1 h and quenching in water	0	175 °C/24 h	A1	521	556	8.8%
	0	155 °C/64 h	A2	527	560	11.3%
	5%	155 °C/24 h	PA	566	596	11.2%
	5%	150 MPa + 155 °C/24 h	PCA	585	604	11.8%
	5%	120 °C/12 h+ 155 °C/12 h	PDA	609	629	11.5%

**Table 3:** Fitted values of parameters from the EEC.

	A1	A2	PA	PCA	PDA
R_s_ (Ω/cm^2^)	3.76	6.12	7.11	8.51	10.03
R_o_ (Ω/cm^2^)	346	466	548	688	776
R_c_ (Ω/cm^2^)	2612	4938	6316	7602	7964
R_p_ (Ω/cm^2^)	2139	2705	3687	4776	5077
C_o_ (μF/cm^2^)	4.33	3.45	3.38	2.96	2.92
C_c_ (10^−4^F/cm^2^)	1.73	1.53	1.40	1.32	1.29
C_p_ (μF/cm^2^)	3.72	3.53	3.20	2.93	2.82
L (10^3^H/cm^2^)	3.20	3.63	5.83	7.15	9.49

**Table 4:** Chemical composition of intermetallic particles in Figure 5 (wt%).

Point	Al	Cu	Fe	Ag	Mg
1	70.66	21.91	6.59	0.84	–
2	67.68	24.81	7.20	–	0.31
3	79.96	14.93	5.11	–	–
4	71.20	20.34	7.73	0.74	–
5	68.98	21.35	9.67	–	–
